# Investigation of Antimicrobial Peptide Genes Associated with Fungus and Insect Resistance in Maize

**DOI:** 10.3390/ijms18091938

**Published:** 2017-09-15

**Authors:** Joseph Noonan, William Paul Williams, Xueyan Shan

**Affiliations:** 1 Department of Biochemistry, Molecular Biology, Entomology and Plant Pathology, Mississippi State University, Mississippi State, MS 39762, USA; jn377@msstate.edu; 2 USDA-ARS, Corn Host Plant Resistance Research Unit, Mississippi State, MS 39762, USA; paul.williams@ars.usda.gov

**Keywords:** plant antimicrobial peptides, defensin, hevein, snakin, cyclotide, maize, host plant resistance

## Abstract

Antimicrobial peptides (AMPs) are small defense proteins present in various organisms. Major groups of AMPs include beta-barrelin, hevein, knottin, lipid transfer protein (LTP), thionin, defensin, snakin, and cyclotide. Most plant AMPs involve host plant resistance to pathogens such as fungi, viruses, and bacteria, whereas a few plant AMPs from the cyclotide family carry insecticidal functions. In this research, a genome-wide investigation on antimicrobial peptide genes in maize genome was conducted. AMPs previously identified from various plant species were used as query sequences for maize genome data mining. Thirty-nine new maize AMPs were identified in addition to seven known maize AMPs. Protein sequence analysis revealed 10 distinguishable maize AMP groups. Analysis of mRNA expression of maize AMP genes by quantitative real-time polymerase chain reaction (qRT-PCR) revealed different expression patterns in a panel of 10 maize inbred lines. Five maize AMP genes were found significantly associated with insect or fungus resistance. Identification of maize antimicrobial peptide genes will facilitate the breeding of host plant resistance and improve maize production.

## 1. Introduction

Plants are constantly under attack by insect pests as well as bacterial, viral, and fungal pathogens. Various plant defense mechanisms exist to build up a complex and efficient innate plant immune system. The innate plant defense system includes the constitutive defense, which is the primary form of surveillance to provide first-line protection from pathogens, and the induced defense, which is triggered through signal transduction and defense gene expression upon insect feeding or pathogenic infection. The induced plant defense system responds to the pathogenic infection and promotes the production of defense-related secondary metabolites and pathogenesis-related proteins (PR proteins). Plant AMPs are classified as PR proteins with major types including beta-barrelins, heveins, knottins, lipid transfer proteins (LTPs), thionins, defensins, snakins, and cyclotides [1,2,3,4]. The types of plant AMPs present in a plant vary from species to species. Reports of plant AMPs have represented a few plant families, such as Amaranthaceae, Andropogoneae, Brassicaceae, Oryzeae, Solanaceae, Triticeae, and Violaceae [5,6,7,8].

Plant AMPs are peptides or low molecular weight proteins either constitutive or induced to deliver attacks to plant pathogens. Most plant AMPs identified to date demonstrated resistance to fungi, viruses, and bacteria, with only a few AMPs from the cyclotide family showing growth inhibition effects to insect larvae [2,9,10,11,12]. Plant AMPs eliminate pathogens by penetrating and interfering with the structural components of cell membranes. Many plant AMPs function by embedding themselves in the microbial cell membrane and forming pore-like membrane openings that cause leakage of essential ions and nutrients which ultimately leads to cell death [1,12,13]. Plant AMPs possess common characteristics and conserved structures, featuring a characteristic cysteine-rich motif. The conserved cysteine residues are the sites to form multiple disulfide bonds. They are generally peptides or small proteins having less than 90 amino acids in length. Many of them are basic proteins, carrying net positive charges in physiological neutral cellular environments. Despite the highly conserved protein structures, the genes coding for plant AMPs are highly polymorphic. In general, they are genes encoded by two or three exons, with each exon encoding for a different domain. Only the cysteine-rich domains will be present in the mature protein [1,2,3,14,15]. The unique characteristics and conserved structures of AMPs allow them to be easily recognized and grouped into different AMP families. While most are linear, cyclic AMPs exist including bacteriocins in bacteria, theta-defensins in animals, and cyclotides in plants. Many AMPs are rich in cysteine residues which function to stabilize the tertiary structure of these peptides through the cross-bracing of multiple disulfide bonds. The resulting compact structures exhibit high thermal, chemical, and enzymatic stability. Many linear antimicrobial peptides are near cyclic due to their multiple disulfide bonds [1,11]. Their subsequent compact structures similarly result in high thermal, chemical, and enzymatic stability. The sequences of plant AMPs identified to date are available from the databases UniProt and PhytAMP, allowing the search of AMP sequence information and biological data. These databases also provide bioinformatics analysis tools to enhance the understanding of plant AMPs [5,16].

AMPs are classified based on their cysteine motifs, sequence similarity, and conserved secondary and tertiary structures. Plant defensins are an abundant family of plant AMPs. Rich in cysteine residues, plant defensins are cationic proteins between 45–54 amino acids. Triple-stranded antiparallel β-sheets and an α-helix make up the structural conformation essential to the antimicrobial activity of plant defensins. These complex structures are stabilized by 4 to 5 disulfide bridges. Plant defensins have diverse functions including α-amylase and trypsin inhibitory activity in addition to the typical AMP functions. Furthermore, the cationic nature of plant defensins facilitates interaction with sphingolipids and phospholipids of fungal membranes, ultimately disrupting homeostasis [1,17,18,19]. Through an unelucidated mechanisms, retarded cell growth or cell death occurs in fungus cells because of plant defensin interactions. Some proposed mechanisms suggest plant defensins initiate an autophagy-like response, or they induce reactive oxygen species within pathogenic fungi [18]. Heveins and hevein-like peptides are glycine-rich AMPs. They are basic peptides roughly 29–45 amino acids long with 3 to 5 disulfide bonds. Heveins bind to chitin and function to inhibit the growth of chitin-containing fungi thereby conferring defense against a plethora of fungal pathogens [1].

Knottin-type peptides make up a large group including the cyclotide family. Cyclotides are antimicrobial peptides only identified in plants. They are the only class of plant AMPs that demonstrate insecticidal activities [1,5,10,12,20]. Cyclotides are a family of plant AMPs with unique head to tail cyclized peptide structure. With the help of computational analysis and database screening, cyclotides have been identified mainly from Rubiaceae, Violaceae and Poaceae families [1,21]. They are small disulfide-rich proteins that have the unusual feature of a cyclic backbone and a cysteine knot. This protein consists of three disulfide bonds with connecting backbone segments which forms a ring in the structure that is penetrated by its third disulfide bond. They are among the smallest AMPs, 29–37 amino acids long, and the most diverse in function [1,10,11]. They can have hormone-like functions or activities related to enzyme inhibition, as well as cytotoxic, antimicrobial, insecticidal, and anti-HIV activities [1,10,11,12,22]. Despite the highly conserved motif, knottin-type peptides have hypervariable sequences. Due to the high sequence tolerance and diverse biological function, the knottin scaffold has even been used as a template for drug design. Cyclotides are particularly stabile due to their cyclic cysteine knot motif and cyclization. They are resistant to gastrointestinal proteases like trypsin, chymotrypsin, pepsin, and elastase. In addition, the cyclization results in limited unfolding thereby increasing resistance to elevated temperatures. The resulting topology formed by the cyclized backbone and the cysteine knot make cyclotides a highly unusual and interesting class of protein [1,3,9,12,20,21].

Lipid transfer proteins (LTPs) are cationic proteins roughly 20–25 amino acids long with 4 disulfide bonds. They are particularly known for their lipid transfer activity, binding to a wide variety of lipids. These proteins promote membrane permeability in pathogens rather than host cells. Rich in lysine and arginine, α-hairpinins are plant defense peptides roughly 33 amino acids long. Well documented for antibacterial and antifungal activities, α-hairpinins inhibit spore germination and hyphal elongation of several plant pathogenic fungi in vivo. These are most known for their helix-loop-helix motif. Snakins have six disulfide bonds and are no longer than 66 amino acids. They are a component of the constitutive and inducible plant defense barriers and are known for their efficacious antifungal and antibacterial activities at low concentrations [23].

Plant AMPs are naturally occurring antimicrobial proteins expressed in various plant species. A thorough understanding of the occurrence and distribution of plant AMPs in crops is of great importance to increase crop yield and reduce plant diseases. The objectives of this research are to conduct a genome-wide investigation on maize AMP genes and examine their expression levels using maize inbred lines associated with various fungus and insect resistance. Especially, objectives of this research are to characterize and understand maize AMPs, their potential antimicrobial functions, and the number and distribution of AMPs in maize genome. Identification of maize antimicrobial proteins against agriculturally important insects and fungi will provide great insights and powerful methods for maize protection.

## 2. Results

### 2.1. Identification of Maize AMPs

Plant AMPs from PhytAMP Database [5] were used to BLAST the maize B73 genome database MaizeGDB [24,25]. There was a total of 271 plant AMPs from various plant families including Amaranthaceae, Andropogoneae, Brassicaceae, Oryzeae, Triticeae, and Violaceae curated in the PhytAMP database [5]. Of the 271 sequences, 189 plant AMP sequences were used to BLAST the whole maize B73 genome. Thirteen out of all the plant AMPs from the PhytAMP Database from various species yielded significant hits from the BLAST of whole maize B73 genome sequence (Table 1). These plant AMP sequences included 2 heveins from *Beta vulgaris* and *Eutrema wasabi*, respectively, 3 snakins each from *Fagus sylvatica*, *Fragaria ananassa*, and *Zea mays*, respectively, 2 LTPs from *Zea mays*, 2 defensins from *Arabidopsis thaliana*, a defensin from *Zea mays*, a beta-barrelin from *Macadamia integrifolia*, and 2 cyclotides from *Chassakia parviflora* and *Viola hederacea*, respectively (Table 1). A total of 46 maize protein sequences with homology to known plant AMPs were identified (Table 1). This included 39 new maize AMPs and 7 known maize AMPs. In some cases, 2 to 3 maize AMP sequences were found to derive from the same gene which produces variable lengths of protein sequences, such as GRMZM2G005633P1 and GRMZM2G005633P2 (Table 1). All of these variable versions appeared to be full length and were located to the same chromosome loci, with some of these loci close to transposable elements. All these sequences were considered as full-length maize AMPs and were included in further analysis. This resulted in the identification of 8 maize heveins, 12 maize snakins, 7 maize LTPs, 15 maize defensins, 1 maize beta-barrelin, and 3 maize cyclotides. Therefore, the B73 genome contains six potential AMP families. The names of the identified maize AMPs followed the Gramene ID system from MaizeGDB (Table 1). Maize AMPs were found on each of the 10 maize chromosomes (Table 1). The protein sequences of maize AMPs ranged from 35 aa (defensin GRMZM2G153488) to 379 aa (hevein GRMZM2G145528). The protein sequences of all the identified maize AMPs were summarized in Table S1.

### 2.2. Phylogenetic Analysis and Protein Motif Detection of Maize AMPs

**Figure 1:**
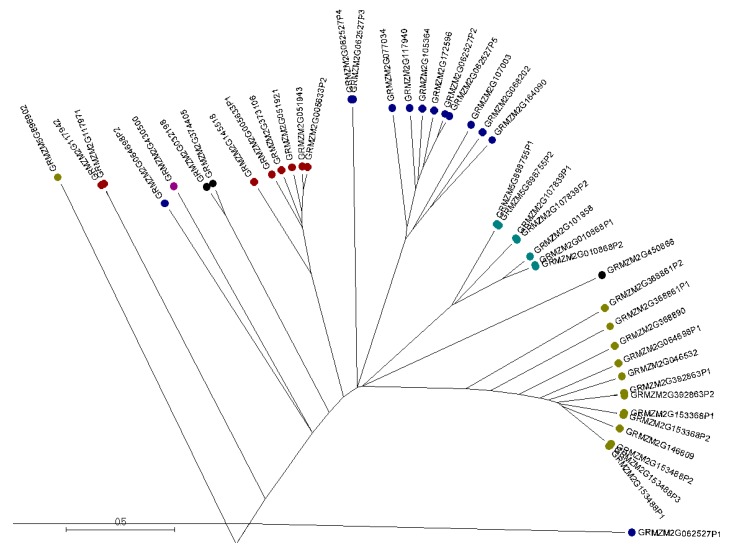
A phylogenetic tree of maize antimicrobial peptides. The forty-six identified maize AMPs are separated into 10 groups. Color coding indicates the corresponding AMP families with Hevein (red), Snakin (blue), Defensin (yellow-green), cyclotide (black), beta-barrelin (purple), and lipid transfer protein (LTP) (aqua). The phylogenetic tree is displayed with software MEGA 7.

**Figure 2:**
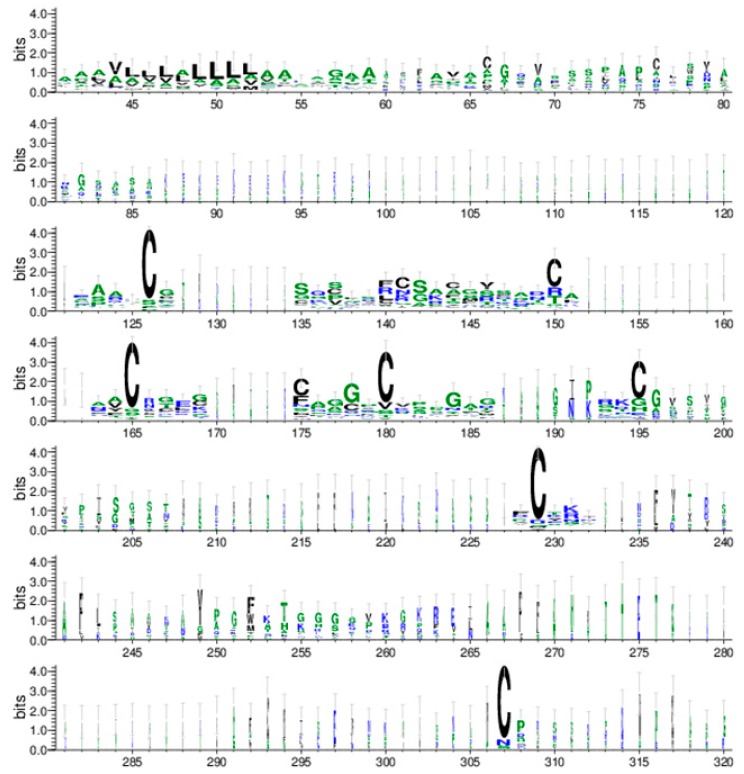
A WebLogo graph showing the positions of highly conserved cysteine-rich motifs observed among all forty-six maize AMP sequences. The degree of conservation is indicated by the relative size of the single-letter amino acid code.

Multiple sequence alignments of the 46 maize AMPs were generated by using the software Muscle [26,27] and displayed by MSA software (http://msa.biojs.net/) (Figure S1). The phylogenetic tree of the maize AMP families was constructed with Muscle and displayed with MEGA7 [28]. The maize AMPs appeared to be classified into 10 groups (Figure 1). Most of the maize AMPs fell into the predicted six groups including hevein, snakin, LTP, defensin, beta-barrelin, and cyclotide. Some of the maize AMPs, such as GRMZM2G062527P3, GRMZM2G062527P4, GRMZM2G117942, and GRMZM2G117971, further separated into more groups. To validate whether the identified maize AMPs carried the characteristic cysteine-rich motifs, each protein sequence was evaluated by using the bioinformatics web-server Pfam [29]. Each of these maize genes were verified to contain sufficient sequence similarity to cysteine-rich motifs found within known AMP models according to the Pfam web-server. These maize AMPs were then grouped into appropriate AMP gene families based on Pfam results (data not shown). The web-server software WebLogo [30,31] was used to further illustrate the characteristic cysteine-rich motif of these maize AMP sequences (Figure 2). Highly conserved amino acids within the WebLogo arrangement are depicted as relatively larger single letter code characters. From the WebLogo software, this arrangement reveals the highly conserved cysteine residues within the maize sequences across all groupings. The nature of this highly conserved cysteine-rich motif implies they have the capability of forming the characteristic multiple disulfide bonds found within AMPs (Figure 2, Supplemental Material). In addition to these findings, a conserved leucine-rich motif was observed. This motif preceded the cysteine-rich motif in the WebLogo graph (Figure 2).

### 2.3. Expression Analysis of Maize AMP Genes and Polymorphism Study of Their Genomic Sequences

**Figure 3:**
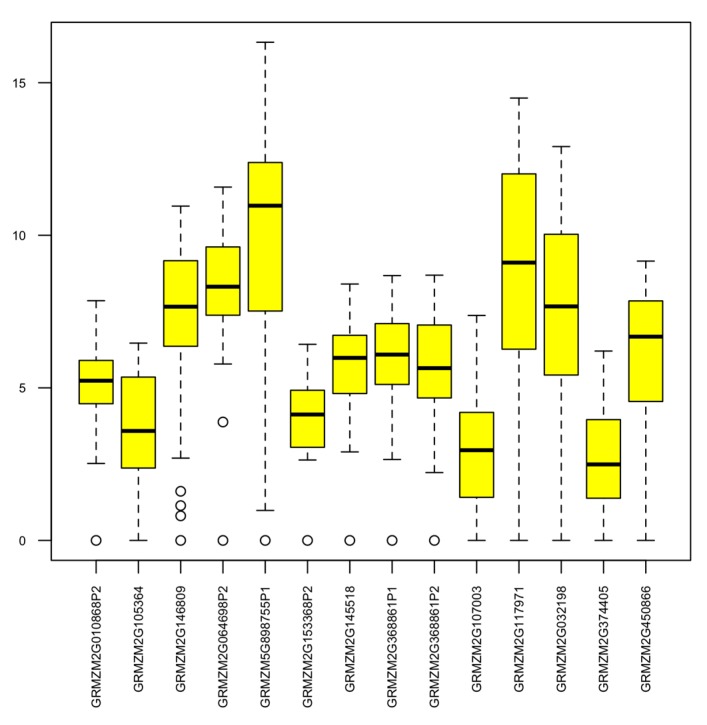
A boxplot showing the statistical summary of the range of relative gene expression levels of selected maize AMP genes across all 10 maize inbred lines.

**Figure 4:**
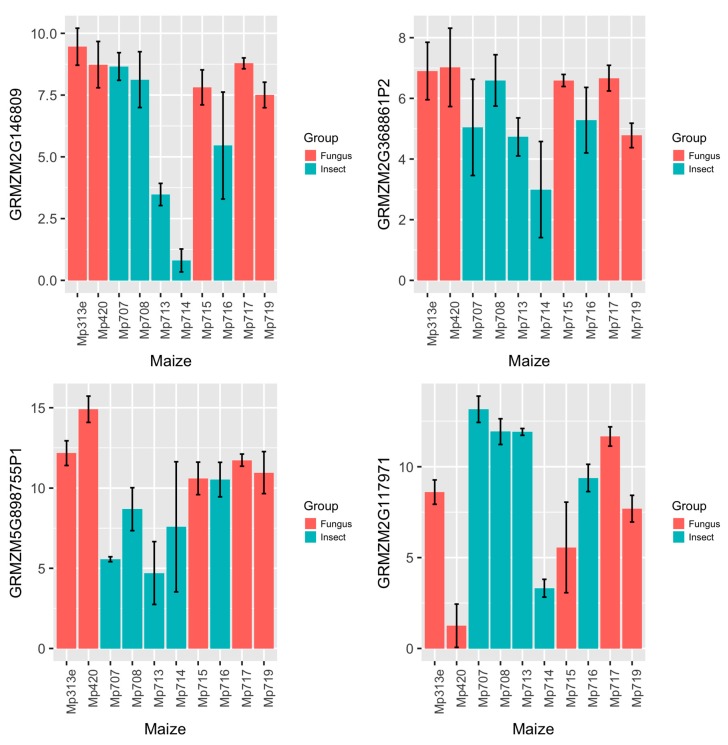
Bar graphs showing the relative gene expression levels in maize inbred lines that grouped by fungus resistance or insect resistance. The qRT-PCR data revealed variations in gene expression levels of the maize AMPs with distinct maize fungus and insect resistance groups. The expression of GRMZM2G146809 (a defensin on maize chromosome 10) reveals a dichotomy between the two sets of maize inbred lines. Relative Expression of Lipid Transfer Protein GRMZM5G898755 (located on maize chromosome 10) displayed significant variations across the 10 maize inbred lines. The relative expression of GRMZM2G368861 (located on maize chromosome 10) showed similar patterns associated with fungal resistance. Expression of maize Hevein GRMZM2G117971 (on maize chromosome 4) varied greatly across the 10 maize inbred lines and averaged higher in expression associated with insect resistance.

**Figure 5:**
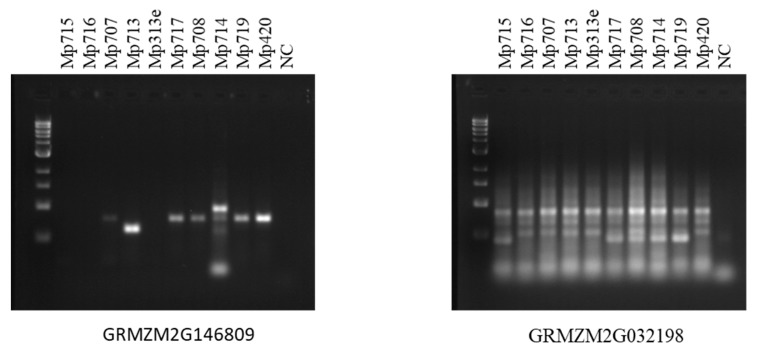
An agarose gel image showing DNA polymorphisms for two maize AMP genes. Gene specific DNA markers were designed for the differentially expressed maize AMPs from maize genomic DNA in order to promote marker-assisted breeding application. DNA polymorphisms were observed among the 10 maize inbred lines in defensin GRMZM2G146809 and cyclotide GRMZM2G032198.

To determine the functions of maize AMPs, the gene expression levels of maize AMPs were investigated by qRT-PCR using 10 maize inbred lines carrying different levels of fungus or insect resistance. Five of the maize inbred lines (Mp313e, Mp420, Mp715, Mp717, and Mp719) are resistant to the fungus *Aspergillus flavus* but susceptible to insect pests such as southwestern corn borer and fall armyworm. The other five maize inbred lines (Mp707, Mp708, Mp713, Mp714, and Mp716) carry insect resistance but fungus susceptibility. These maize inbred lines are not all derived from the same pedigrees. Various molecular defense pathways may exist while common resistance mechanism may also be possible. To identify which maize AMPs were involved with the observed resistance, maize leaf samples were collected from field corn plants in a randomized experimental design with three replications for each maize inbred line. Figure 3 displays a boxplot showing the summary of gene expression data from a group of 14 AMP genes that represented maize hevein, defensin, snakin, LTP, and cyclotide groups. The qRT-PCR data revealed variations in gene expression levels of the maize AMPs. The qRT-PCR data was subjected with statistical analysis by SAS program. Five maize AMPs were found significantly different in gene expression levels between distinct maize fungus and insect resistance groups. Figure 4 shows bar graphs for four of the significant differentially expressed maize AMP genes. The observed expression of GRMZM2G146809 (a defensin on maize chromosome 10) reveals a dichotomy between the two sets of maize inbred lines color-grouped according to their distinct types of resistance. Within the set of maize inbred lines associated with fungal resistance and insect susceptibility, the overall expression of GRMZM2G146809 was observed to be significantly greater than the insect resistant and fungal susceptible set of maize inbred lines (*p* = 0.01). Relative Expression of Lipid Transfer Protein GRMZM5G898755 (located on maize chromosome 10) displayed the widest range across the 10 maize inbred lines (Figure 3). The highest expression was observed in Mp420, fungal resistant, and the lowest expression was observed in Mp713, insect resistant (Figure 4). Additionally, the mean ∆Cp of each maize inbred line within the set associated with fungal resistance and insect susceptibility was greater than those associated with insect resistance and fungus susceptibility. The relative expression of GRMZM2G368861 (located on maize chromosome 10) showed similar patterns associated with fungal resistance. Although the expression of GRMZM2G368861 between the two sets of maize inbred lines with contrary resistance type was significantly different, there was less variation in the overall expression of GRMZM2G368861 within the groups. Expression of maize Hevein GRMZM2G117971 (on maize chromosome 4) varied greatly across the 10 maize inbred lines. The highest mean value was associated with insect resistance. Although insect resistant Mp714 displayed the lowest relative expression, different insect resistance mechanisms may exist among the maize inbred lines. This result makes hevein GRMZM2G117971 the only significant maize AMP reported here to be expressed significantly greater in the group of maize inbred lines associated insect resistance and fungal susceptibility. Gene specific DNA markers were designed for the differentially expressed maize AMPs from maize genomic DNA in order to promote marker-assisted breeding application. DNA polymorphisms so far were observed among the 10 maize inbred lines including defensin GRMZM2G146809 and cyclotide GRMZM2G032198 (Figure 5).

## 3. Discussion

The most prominent characteristic of AMPs is their structural topology. This circular, or near circular, topology allows AMPs to withstand extreme proteolytic environments [1,4,12,20]. For example, the topology of cyclotides confers such a high degree of stability that a harsh microwave-based extraction methodology can be found in literature [32]. Although many families of AMPs can be differentiated by their structural topologies, plant AMPs are largely classified by their highly conserved cysteine-rich motifs [1,2,3,11]. Similar observations were confirmed from the identified maize AMPs by using multiple sequence alignment analysis subjected to the HMM pattern characterization algorithms (Figure 2 and Supplemental Material) [33]. Using Pfam, each maize sequence was characterized with cysteine-rich motifs. The WebLogo software further elucidated these motifs. From this arrangement, the highly conserved cysteine residues were distinguishable thereby implicating which residues can form multiple disulfide bonds (Figure 2, Supplemental Material). The antimicrobial activity of AMPs is dependent upon these disulfide bonds [2].

Despite the well-documented roles of plant AMPs in antimicrobial resistance, the biological functions of many plant AMPs remain largely unknown [1,2,3,11,34]. The fundamental principle underlying popular theories regarding AMPs and their mechanism of action revolves around the penetration and aggregation of AMPs into the microbial membrane of pathogens [1,2,3,12]. This penetration is facilitated by their amphipathic nature, a commonality among cysteine-rich proteins [1,2,3,35]. This feature is especially true for cyclotides as they are correlated with hydrophobicity [1,2,3,4,8,9]. The interaction of AMPs with these microbial membranes results in the formation of pore-like structures that are conducive to cell lysis through unregulated influx/efflux of essential ions.

Interestingly, a hydrophobic patch preceding the cysteine-rich motif was observed using the WebLogo software. Although literature reports hydrophobic patches are involved with the penetration of the microbial cell membrane [1,9,10,14,22,32], a leucine-rich motif was observed in the maize sequences. In addition to these conserved leucine residues, other hydrophobic residues were observed in this hydrophobic patch, namely alanine and to a lesser extent valine. Prior to this finding, no literature reports highly conserved leucine residues within the hydrophobic patches of AMPs. As a result, a leucine-rich motif may be characteristic of hydrophobic patches in maize AMPs.

Although many AMPs are a part of the constitutive defense [1,2,3,7,10,36], insecticidal proteins were of significant interest in this study due to the relatively limited literature available. To investigate these oftentimes inducible AMPs, only plant tissue that was directly wounded by insect feeding was collected. Some AMPs, such as snakin-2, are both constitutive and locally expressed in response to wounding [1,34]. Others have also been reported to be expressed relative to the release of hormones, such as thionin in response to methyl jasmonate [1,6,7,37]. Therefore, the wounding of plant tissue prior to collection was considered essential for the relative expression analysis of AMPs within the panel of maize inbred lines analyzed in this study.

Out of the 189 plant AMPs reviewed from the PhytAMP database, 13 plant AMP sequences representing six AMP gene families that originated from nine plant species were used to identify sequences with high similarities in Maize B73 genome. Thirty-nine new maize AMPs were identified in this study in addition to 7 known maize AMPs documented in the PhytAMP database. All 46 maize AMPs fall into 10 groups by phylogenetic analysis, with six major groups in accordance to the AMP families of hevein, snakin, LTP, defensin, beta-barrelin, and cyclotide (Figure 1). Four other maize AMP groups each constitutes one or two maize AMPs were also identified at positions apart from the major maize AMP groups which separated earlier on the phylogenetic tree (Figure 1). Interestingly, maize AMPs were found to distribute on each of the 10 maize chromosomes with some loci located close to maize transposable element active regions. Given the vast antimicrobial functions of plant AMPs [1,2,5,11], the genome-wide investigation of maize AMPs is of great importance to reveal naturally occurring maize resistance genes for elimination of plant diseases and improvement of maize production.

Out of the five original Plant AMPs that identified five significant maize AMPs associated with either fungus resistance or insect resistance, Q8H950 from *Eutrema wasabi* (maize counterpart GRMZM2G117971) was listed with antibacterial and antifungal functions in the PhytAMP Database, P56879 from *Chassalia parviflora* (maize counterpart GRMZM2G032198) was listed with antibacterial, antifungal, and insecticidal functions (Table 1, Figure 4). P81008 from *Zea mays* is a sodium channel blocker. Four maize AMPs similarly matched this sequence in the maize genome. On the other hand, Q2XX14 from *Zea mays* (with four new maize counterparts, including GRMZM2G898755P1) and Q9ZUL7 from *Arabidopsis thaliana* (maize counterpart GRMZM2G146809) were previously listed with unknown functions. In this study, we performed a gene expression analysis of maize AMP genes. Using a panel of 10 maize inbred lines carrying contrast levels of fungal or insect resistance, this study provided a gateway method to identify the potential functions of maize AMPs in plant defense systems.

Maize genome database data-mining allowed the identification of 46 maize AMP gene sequences. The number of maize AMP sequences varied from one in the beta-barrelin family to 13 in the defensin family. This is largely similar to the amount and types of AMPs distributed in other plant species. The maize AMP genes were found to distribute across the maize genome. One to three different maize AMP families were found on each maize chromosome (Table 1). The phylogenetic tree revealed, in many cases, that two or three maize AMP genes clustered together due to the presence of shorter versions of protein sequences derived from the same maize AMP gene (Figure 1). This indicated that alternative mRNA slicing mechanisms may exist in these maize AMP genes. The functional implications of such arrangements of the AMP genes in maize genome will be the subjects of future studies. In summary, our study identified 46 maize AMP sequences, four of them showing significance associated with fungal resistance and one showing significance related to insect resistance. This study also revealed polymorphisms in maize AMP genomic sequences that will be valuable for maize breeding applications. Together, these new findings will facilitate the identification of new natural plant resistance sources to confer fungus and insect resistance in maize. Future work based on the identification of maize AMP genes will focus on the extraction and characterization of maize AMPs to gain insights on the mechanisms and the antipathogenic peptide profiles in maize germplasm related to *Aspergillus flavus* and *Spodoptera frugiperda* resistance.

## 4. Materials and Methods

### 4.1. Plant Material

Five maize (*Zea mays*) inbred lines with insect resistance and fungus susceptibility (Mp707, Mp708, Mp713, Mp714, and Mp716) as well as five maize inbred lines with insect susceptibility and fungus resistance (Mp313e, Mp420, Mp715, Mp717, and Mp719) were grown in field plots at the R.R. Foil Plant Science Research Center at Mississippi State University. The insect resistant inbred lines were selected based on the resistance to leaf-feeding by fall armyworm (*Spodoptera frugiperda*). Plants were infested with 30 larvae per maize plant during the mid-whorl stage of growth. Fourteen days after infestation, sections of leaves damaged by insect feeding were cut from maize plants and frozen in liquid nitrogen. Maize leaf samples were ground into powder via motor and pestle with the aid of liquid nitrogen. Samples were then stored at −20 °C.

### 4.2. DNA Extraction

Extraction of plant DNA samples was performed using a DNeasy^®^ Plant Mini Kit from Qiagen^®^ with minor modifications from the manufacturer manual. Approximately 200 mg of ground leaf tissue was transferred to pre-chilled centrifuge tubes using pre-chilled spatulas. In each sample tube, 400 µL of buffer AP1 and 4 µL RNase A were added. These were vortexed twice and incubated in a water bath at 65 °C for 5 min. The lysate was centrifuged for 1min at 12,000 rpm and the supernatant was transferred to a fresh tube. The rest of the extraction was performed per the manufacturer protocol. Samples were then quantified using a Nanodrop2000c spectrophotometer (Thermo Fisher Scientific, Wilmington, DE, USA). The purity of the products was assessed via their A_260_/A_280_ values. DNA samples were stored at −20 °C.

### 4.3. Database Search for Maize (Zea mays) Antimicrobial Peptide Gene and Protein Sequences

Maize antimicrobial peptide sequences were obtained using various databases. First, plant AMP sequences from various species in the PhytAMP Database (phytamp.pfba-lab-tun.org/) were used to search the UniProt protein database (uniport.org/). Amino acid and nucleic acid sequences were collected from the seven superfamilies—Cyclotide, Defensin, Hevein, Knottin, Lipid-transfer protein, Snakin, and Thionins based on the classifications in the PhytAMP Database. The basic alignment search tool (BLAST) was used to search for similar antimicrobial peptide sequences in MaizeGDB B73 genome database (maizegdb.org/). The corresponding nucleic acid sequence was acquired in addition to the amino acid sequence and their physical locations on maize chromosomes were also determined from MaizeGDB B73 genome database. Each protein sequence was then subjected to Pfam for motif identification (pfam.xam.org/). Regions containing AMP domains were later used for primer design.

### 4.4. Design of Genomic DNA and cDNA Primers for Maize AMPs

Nucleic acid sequences gathered from MaizeGDB gene regions containing AMP domains were used as templates for primer design. The Primer3 online software (http://bioinfo.ut.ee/primer3-0.4.0/primer3/) was used to design primers. Primers were designed such that product size ranged between 400 and 800 bp for genomic DNA. The cDNA sequence representing the mature mRNA sequence for the gene of interest was used as the template for the design of primers used in quantitative real-time polymerase chain reaction (qRT-PCR). cDNA primers were designed using the primer3 online software such that products were approximately 150 bp. DNA oligos were synthesized by Sigma Life Sciences (Sigma-Aldrich, Inc., St. Louis, MO, USA) (Table 2).

### 4.5. Polymerase Chain Reaction for Genomic DNA

One microliter of DNA sample was added into 24 µL of PCR reaction mixture containing 1× ThermoPol Reaction Buffer from New England BioLabs (New England BioLabs, Inc., Ipswich, MA, USA), 200 µM dNTP from New England BioLabs, 1 µM forward primer, 1 µM reverse primer, and 2.5 units Taq polymerase/50 µL PCR. For each set of reaction mixtures, 1 µL of ddH_2_O was used as a control. Upon starting, the thermocycler held an initial denaturation for 3 min at 95 °C. Afterwards, the PCR was performed with a denaturation step for 45 s at 94 °C, an annealing step of 50 s at 52 °C, and an elongation step at 72 °C for 80 s for 40 cycles. A final extension lasted for 10 min at 72 °C before a final hold step at 4 °C. Amplification of PCR products was determined via agarose gel electrophoresis using 1% (*w*/*v*) agarose in 1× TAE buffer. 1 kb DNA ladder from New England BioLabs (New England BioLabs, Inc., Ipswich, MA, USA) was used. The electrophoresis was conducted at 75 V on constant voltage for 75 min. Agarose gels were then stained with ethidium bromide for 60 min and de-stained in water for 10–60 min prior to viewing with a UV gel-imager.

### 4.6. RNA Extraction

RNA was extracted using the spin column protocol with minor modifications according to the Aurum™ Total RNA Fatty and Fibrous Tissue Kit from Bio-Rad (Bio-Rad Laboratories, Inc., Hercules, CA, USA). To start the extraction, frozen leaf tissue was ground to a fine powder using mortar and pestle with liquid nitrogen. In 2.0 mL microfuge tubes, 100 mg of tissue was suspended in 1 mL PureZOL, vortexed for 1 min, and incubated at room temperature for 5 min. Samples were then pelleted at 12,000× *g* for 5 min at 4 °C. After transferring the supernatant to new 2.0 mL tubes, 200 µL of chloroform was added to the lysate. The sample was then centrifuged at 12,000× *g* for 15 min at 4 °C. The aqueous phase was then transferred to new 2.0 mL tubes without disturbing the interphase. Equal volume 70% ethanol was then added to the sample and mixed via pipetting. Then the manufacturer protocol was followed to finish the extraction. A volume of 30× of the elution buffer was used to elute each RNA sample via centrifugation for 2 min at 12,000× *g*.

### 4.7. Synthesis of cDNA and qRT-PCR Analysis

The synthesis of cDNA was completed using the ThermoScript^TM^ RT-PCR System kit from Life Technologies (Life Technologies Corporation, Carlsbad, CA, USA) and the manufacturer protocol was followed. The RNA was denatured at 65 °C for 5 min and then held at 4 °C. The synthesis of cDNA was carried out at 50 °C for 60 min, 85 °C for 5 min, and then held indefinitely at 4 °C. The cDNA samples were then stored in −20 °C. For qRT-PCR, the LightCycler^®^ 480 SYBR Green I Master enzyme mix (Roche Diagnostics Corporation, Indianapolis, IN, USA) was used. The run protocol contained one denaturation step at 95 °C for 5 min, followed by 45 cycles of 95 °C for 10 s, 60 °C for 15 s, and 72 °C for 15 s. The final step was a 10 s cool down to 40 °C. To determine the efficiency of the amplification, primer efficiency was evaluated by diluting cDNA using a five-step 3-fold serial dilution. The coefficient of determination (*R*^2^) from the linear regression of ΔCp and Log(1/dilution factor) was retrieved. Following this, the efficiency was calculated by Log_2_(*r* + 1), where *r* = *R*^2^. Primers with the efficiency above 0.7 were used for qRT-PCR analysis of gene expression levels on the ten maize inbred lines. The expression level of GAPDH gene from each sample was evaluated and used as a house-keeping control gene for normalization purpose.

### 4.8. Statistical and Bioinformatics Analysis

For qRT-PCR analysis of gene expression, the Cp values were normalized and used for statistical analysis. Amplification efficiencies of cDNA primers were incorporated to calculate the Cp values. The expression level of glyceraldehyde 3-phosphate dehydrogenase (GAPDH) was used as a house-keeping gene control for the data normalization. SAS University Edition for Windows (SAS Institute Inc., Cary, NC, USA) was used to conduct ANOVA analysis for gene expression data. Least Significant Difference tests (LSDs) at *p* < 0.05 and *p* < 0.01 levels were performed for estimate of the significance of differential gene expression. Statistics results were graphed with the R package ggplot2 (http://ggplot2.org/). Multiple sequence alignments were generated by using the software Muscle [23,24] and displayed by MSA (http://msa.biojs.net/). The phylogenetic tree was constructed with Muscle and displayed with Mega 7 [25]. To validate the characteristics of the maize AMP motifs, the bioinformatics web-server Pfam was used [26]. The web-server software WebLogo [27,28] was used to illustrate the characteristic protein motifs.

## Figures and Tables

**Table 1:** Maize antimicrobial peptides (AMP) genes identified by basic alignment search tool (BLAST) maize B73 genome with known plant AMP sequences from various species.

Maize Gene ID	Maize Chromosome	BLAST From Seq	Origin
Hevein			
GRMZM2G005633P1	Ch10	ctb1-chitinase B1	*Beta vulgaris*
GRMZM2G005633P2	Ch10	ctb1-chitinase B1	*Beta vulgaris*
GRMZM2G373106	Ch8	ctb1-chitinase B1	*Beta vulgaris*
GRMZM2G117942	Ch4	Q8H950	*Eutrema wasabi*
GRMZM2G145518	Ch6	Q8H950	*Eutrema wasabi*
GRMZM2G051921	Ch2	Q8H950	*Eutrema wasabi*
GRMZM2G051943	Ch2	cta1-chitinase	*Beta vulgaris*
GRMZM2G117971	Ch4	Q8H950	*Eutrema wasabi*
Snakin			
GRMZM2G105364	Ch9	Q0VYL5	*Fagus sylvatica*
GRMZM2G068202	Ch2	Q19VG5	*Zea mays*
GRMZM2G117940	Ch1	O49134	*Fragaria ananassa*
GRMZM2G172596	Ch10	Q0VYL5	*Fagus sylvatica*
GRMZM2G062527P1	Ch6	Q19VG5	*Zea mays*
GRMZM2G062527P2	Ch6	Q19VG5	*Zea mays*
GRMZM2G062527P3	Ch6	Q19VG5	*Zea mays*
GRMZM2G062527P4	Ch6	Q19VG5	*Zea mays*
GRMZM2G062527P5	Ch6	Q19VG5	*Zea mays*
GRMZM2G077034	Ch5	O49134	*Fragaria ananassa*
GRMZM2G164090	Ch6	Q19VG5	*Zea mays*
GRMZM2G107003	Ch2	Q19VG5	*Zea mays*
LTP			
GRMZM2G010868P1	Ch3	Q2XX14	*Zea mays*
GRMZM2G010868P2	Ch3	Q2XX14	*Zea mays*
GRMZM2G101958	Ch10	Q2XX14	*Zea mays*
GRMZM5G898755P1	Ch10	Q2XX14	*Zea mays*
GRMZM5G898755P2	Ch10	Q2XX14	*Zea mays*
GRMZM2G107839P1	Ch3	Q2XX25	*Zea mays*
GRMZM2G107839P2	Ch3	Q2XX25	*Zea mays*
Defensin			
GRMZM2G368890	Ch10	P81008	*Zea mays*
GRMZM2G392863P1	Ch1	Q9ZUL7	*Arabidopsis thaliana*
GRMZM2G392863P2	Ch1	Q9ZUL7	*Arabidopsis thaliana*
GRMZM2G146809	Ch10	Q9ZUL7	*Arabidopsis thaliana*
GRMZM5G896902	Ch2	Q9ZUL7	*Arabidopsis thaliana*
GRMZM2G153488P1	Ch5	P82781	*Arabidopsis thaliana*
GRMZM2G153488P2	Ch5	P82781	*Arabidopsis thaliana*
GRMZM2G153488P3	Ch5	P82781	*Arabidopsis thaliana*
GRMZM2G064698P1	Ch2	Q9ZUL7	*Arabidopsis thaliana*
GRMZM2G064698P2	Ch2	Q9ZUL7	*Arabidopsis thaliana*
GRMZM2G153368P1	Ch5	P82781	*Arabidopsis thaliana*
GRMZM2G153368P2	Ch5	P82781	*Arabidopsis thaliana*
GRMZM2G368861P1	Ch10	P81008	*Zea mays*
GRMZM2G368861P2	Ch10	P81008	*Zea mays*
GRMZM2G046532	Ch7	P81008	*Zea mays*
Beta-Barrelin			
GRMZM2G430500	Ch2	P80915	*Macadamia integrifolia*
Cyclotide			
GRMZM2G032198	Ch3	P56879	*Chassalia parviflora*
GRMZM2G374405	Ch3	P56879	*Chassalia parviflora*
GRMZM2G450866	Ch7	P85233	*Viola hederacea*

**Table 2:** Polymerase chain reaction (PCR) primers for cDNA and genomic DNA of maize AMP genes.

Maize Genes	Primers
**cDNA Primers**	
Hevein	
GRMZM2G117942F	TACATCGATCGGTTGCCAAA
GRMZM2G117942R	TTCTGCTGCGGGTTGTAGA
GRMZM2G145518F	TTCTCCAAGCACAGGAGACA
GRMZM2G145518R	ACGCCTCACTTCCCACTGTA
GRMZM2G117971F	TATGGATGTGATCCCACACG
GRMZM2G117971R	AGTGGACGACACATATTCGAGA
Snakin	
GRMZM2G105364F	TGGAATGCTACCAGCCAGAT
GRMZM2G105364R	CGGGATGTTCCTCATCAATC
GRMZM2G172596F	CTGCTCCTCTGCTTCCTGTT
GRMZM2G172596R	GTTCTTGTAGCCCTCGTGCTT
GRMZM2G107003F	CGCCACGTTTTGTATGATCC
GRMZM2G107003R	ACACAGACCCATCAACGTCA
LTP	
GRMZM2G101958F	CATATGTGACCGTGTGTTCCA
GRMZM2G101958R	CTCGCCCAGCTTTGTTTTAT
GRMZM2G010868P1F	TTGGCACCAAGCACTAAAGA
GRMZM2G010868P1R	TCCCAAATCATCCCCTAGAA
GRMZM2G010868P2F	CCTGCAACTGCCTCAAGAAC
GRMZM2G010868P2R	TGCATGCATACTACCCTACCTG
GRMZM5G898755P1F	TCGACTGCACCAAGATCAAC
GRMZM5G898755P1R	TCTGATGCATGACACACACG
GRMZM5G898755P2F	AGCAGCACCTCAATGTCCTT
GRMZM5G898755P2R	CATGCATATGTACGGCGAAT
GRMZM2G107839P1F	CTCCGGTTTGCAGAAACAAC
GRMZM2G107839P1R	CTAGGCATCAGCACAGTCCA
GRMZM2G107839P2F	GATCCACCTACTTGTTCAGACAG
GRMZM2G107839P2R	CATCTCCTCTGATCGTCCTTT
Defensin	
GRMZM2G368890F	GCCGGAATATGTGGACGAT
GRMZM2G368890R	ACATGCAGACCCCCTTGAA
GRMZM2G392863P1F	TGTTGTACGTACGTCTGCCTCT
GRMZM2G392863P1R	AACAATCAGCGTCGTCTCTT
GRMZM2G392863P2F	CCGCTGAGATCCTAGGAAGA
GRMZM2G392863P2R	CTGATGAGTCCACAGCACAGA
GRMZM2G146809F	GGTCCGTTTGCGTTTGTTTC
GRMZM2G146809R	GGTTCATCAATGCAACGAGAC
GRMZM5G896902F	AGAAGGACAGCGAGCGATT
GRMZM5G896902R	CCGGGAGTAGGTTAATTTAGCA
GRMZM2G153488P1F	GTTGTACTTTCTGCATCCGTTG
GRMZM2G153488P1R	TTGGTCATCAAGTTCCCTAGC
GRMZM2G153488P2F	AGCCTTACGTAGCGAAGCTC
GRMZM2G153488P2R	AGCAACGAGGAGTTGAGTCG
GRMZM2G153488P3F	ATAAACCGTGGCTCTGGTTC
GRMZM2G153488P3R	TTGCTCTGAGCTTCGCTACG
GRMZM2G064698P1F	AGTTCGTGAATCCCTGAAGC
GRMZM2G064698P1R	ATTCCCTTGCCTGTGCCATA
GRMZM2G368861P1F	GATAGTGACGTACGCGCAAC
GRMZM2G368861P1R	GCATACGATCTGACGCTCAT
GRMZM2G368861P2F	GCGATGGAGCTCATCAAGTC
GRMZM2G368861P2R	GTCCATGAGGCAGCAGAAAT
GRMZM2G046532F	GGTGCCCATACCATAGCTTC
GRMZM2G046532R	TAACAAACGAGCAGGAGGAG
GRMZM2G064698P2F	AGTTCGTGAATCCCTGAAGC
GRMZM2G064698P2R	ATTCCCTTGCCTGTGCCATA
GRMZM2G153368P1F	AAGAAGCCTTGCTAGTTCATCG
GRMZM2G153368P1R	CCCAGCAATTTAAGGACTGC
GRMZM2G153368P2F	GTACGTACTCGTACCAGGCAGA
GRMZM2G153368P2R	GCATGGCTACTCCCATTTTG
β-Barrelin	
GRMZM2G430500F	CTCGGGGGATACGTCGAT
GRMZM2G430500R	TGGGTGTCCTCGAAAACTTG
Cyclotide	
GRMZM2G032198F	GTGTTTGGCCTGGACTTCAT
GRMZM2G032198R	GGCGTCACGAGTTTATTTCA
GRMZM2G374405F	GTCCCCTGTTTTGAATCCTG
GRMZM2G374405R	TTCACACGTAACGGGATCAG
GRMZM2G450866F	GGGCTTGTTGCAGTGGTAGT
GRMZM2G450866R	CGATCTTGTGACGGTTCAGC
**Genomic DNA Primers**	
Defensin	
GRMZM2G146809F2	GGCCAAGTATACTCGCCAGA
GRMZM2G146809R2	TCGAAGGGTTATTGCATTCC
Cyclotide	
GRMZM2G032198F2	GTTGGGAGCAAAGCAAAGAG
GRMZM2G032198R2	GAGGAGCAGGCGATTGAGTA

## Supplementary Materials

Supplementary materials can be found at www.mdpi.com/1422-0067/18/9/1938/s1.
