# Supplementary material for: Investigation of Antimicrobial Peptide Genes Associated with Fungus and Insect Resistance in Maize
Source: Int J Mol Sci. 2017 Sep 15;18(9):1938. doi: 10.3390/ijms18091938 (PMC5618587; doi:10.3390/ijms18091938)
Supplement: Supplementary file 1 [file ijms-18-01938-s001.zip › Antimicrobial Peptide Genes in Maize_Supplemental Table1.pdf]

>GRMZM2G368890

MWTIRKVATPQVAVLLLLLIVVAQEAAPLAEARVCRRRSAGFKGVCMSDHNCAQVCLQEG

YGGGNCDGIMRQCKCIREC

>GRMZM2G368861P1

MELIKSRATVCALLLALLLLSHYDGGTTTTMVAEARVCMGKSQHHSFPCISDRLCSNECV

KEDGGWTAGYCHLRYCRCQKAC

>GRMZM2G368861P2

MELIKSRATVCALLLALLLLSRNKKKHTDLFVDLIHVLFLISAASWTLPPSAIHAQTTTA

GRRRRWWRRPGCAWARASTTRSPASPTASAATSASRRTAGGPPATATSATAGARRRAKQSS

>GRMZM2G046532

MGGLSTKLFVVLLLLVCYTGTQGGPVTMVSARKCESQSFRFKGPCSRDANCANVCLTEGF

TGGVCKGLRHRCFCTRDC

>GRMZM2G392863P1

MELSRKLFTAVLLVMLLLLSAEVGPVAVAEARTCQSQSHRFRGPCLRRSNCANVCRTEGF

PGGRCRGFRRRCFCTTHCH

>GRMZM2G392863P2

MLLRAAAEVGPVAVAEARTCQSQSHRFRGPCLRRSNCANVCRTEGFPGGRCRGFRRRCFC

TTHCH

>GRMZM2G146809

MAWTSRRMVASALVFLLMLLAASEMGTTRVAEARHCTSQSHRFVGACMSKSNCENVCRTE

GFPWGECRWHGIERKCHCKRIC

>GRMZM5G896902

MWRRRRGNARRTASDSLGHAWRRTTAPTCAAVRASPAAGAAPSAAAASALSRAKLTYSRQ
FDGGRLFYLLAYLIFSPLTIRKRTCWHVRCVVYAFLAGFIC

>GRMZM2G153488P1
MALSRRMAAPVLVLMLLLVATELGTTKVAEARHCLSQSHRFKGLCmssNNCANVCQTENF
PGGECKAEGATRKCFCKKIC

>GRMZM2G153488P2
MSSNNCANVCQTENFPGGECKAEGATRKCFCKKIC

>GRMZM2G153488P3
MSSNNCANVCQTENFPGGECKAEGATRKCFCKKIC

>GRMZM2G064698P1
MESSRRFQPAVILLLLLIVSTDMAQARECEKYSERFVGACMIADNCANVCRGEGFLAGRC
STFRRRCICTRQC

>GRMZM2G064698P2
MTNVMYVLVTVMYILMTVMYLVHVCSRFIILFMY

>GRMZM2G153368P1
MAAVSQGAVLFLFLLLVAAEVGTIDAKMGVAMPMHALIMENVKQQEKEKEKEKEEKSTEK
EESRCLSQSLQFEGFCFNSDRCAEVCMKESFPGGECKRDVAMRKCFCKKPC

>GRMZM2G153368P2
MVHAEVGTIDAKMGVAMPMHALIMENVKQQEKEKEKEKEEKSTEKEESRCLSQSLQFEGF
CFNSDRCAEVCMKESFPGGCKRDVAMRKCFCKKPC

>GRMZM2G005633P1

MAMAKAGAPRVSAAQLVTLGLSLLCAVAGPAAAQNCGCQPNVCCSKFGYCGTTDEYCGDG

CQSGPCRSGGGGSSGGGGANVASVVTGSFFNGIKSQAGSGCEGKNFYTRSAFLSAVKAYP

GFAHGGSQVQGKREIAAFFAHATHETGRKLALSINLALQRGAGSLIFDPSC

>GRMZM2G005633P2

MAMAKAGAPRVSAAQLVTLGLSLLCAVAGPAAAQNCGCQPNVCCSKFGYCGTTDEYCGDG

CQSGPCRSGGGGSSGGGGANVASVVTGSFFNGIKSQAGSGCEGKNFYTRSAFLSAVKAYP

GFAHGGSQVQGKREIAAFFAHATHETGHFCYISEINKSNAYCDPTKRQWPCAAGQKYYGR

GPLQISWNYNYGPAGRAIGFDGLGDPGRVARDAVVAFKAALWFWMNSVHGVVPQGFGATT

RAINGALECGGNNPAQMNARVGYYRQYCRQLGVDPGPNLTC

>GRMZM2G373106

MANSATPPPTMILTAALGLTFLLCATTPTAAQHCGCQPGFCCSKYGYCGKTSAYCGEGCK

SGPCWGSAGCGGGGASVARVVTKSFFNGIKSHAGSWCEGRRFYTRSAFLEAIAAYPGFAH

GGSEVERKREIAAFFAHVTHETGHLCYINEVDVAKYCDWSSEKQWPCHPRQGYYGRGPLQ

LSWNYNYGPAGRSLGFDGLGDPDRLAQDPVLSFKSALWYWMENMHQLMPQGFGATIRAIN

GFDECHGGKNTAEMKDRVRFYLEYCHHFRVHPGLDLSC

>GRMZM2G117942

MAGMTVGNKLALAAVLLCAAAAMATAQQASGVRATYNFYNPQQNNWDLNAVSAYCATWDA

SKPLSWRMKYGWTAFCGPAGPTGQAACGQCLLVTNTATGASITVRIVDQCSNGGLDLDYD

TAFKPIDTNGQGFQAGHLTVNYQFVNCGDN

>GRMZM2G145518

MHVIDLSIHASIEMYIYPGSRSHRDHRTRASSLLPMAMTRALAMVAMLATAALFFMSARA

QQCGTQAGGALCPDCLCCSQWGYCGSTPDYCTDGCQSQCFGSGCGGGGGTPATPPSGPVS

EIISESLFNEMLLHRNDVACPAIGFYTYDAFIAAANAFPGFGTTGGADTQKRELAAFLAQ

TSHETTGGWDTAPDGPYTWGYCFKEEVGGVWGPDYCQPSPQWPCADGQKYYGRGPIQLSW

NYNYGPAGEAIGQDLLGNPGLVAADATVSFETALWYWMTPQPPKPSCHDVITGQWAPSPA
DVAAGRLPGYGVLTNIINGGLECGHGADARVASRIGFYKRYCDMFGLSYGDNLDCYDQSP
FGNTILSTTNNTTSYHVDA

>GRMZM2G051921
MVMAMANSATILTVVLALIGLALLRAAAPASAQNDCGSCPPGYCCSKFGYCGTSFDYCNG
NTCQSGPCTAGGAGSGGANVSGVVTDAFLSGIKSQAGSGCEGSSLSFYSRRAFLSAASSY
PGFARAGSEADGKRELAAFFAHVTHETGHFCYVSEVNKNNSYCNSSNTQWPCAPGKKYYG
RGPLQVSWNYNYGPAGRSVGFDGLGNPDMVAQDPVVSFKTALWFWMSNAHQVMPRGFGAT
IRAINGALECNGENPAAVNARVGYYKEYCEQFGVGPGNNLTC

>GRMZM2G051943
MANAPRILALGLLALLCAAAGPAAAQNCGCQPNFCCSKFGYCGTTDAYCGDGCQSGPCRS
GGGGGGGGGGGGGGSGGANVANVVTDAFFNGIKNQAGSGCEGKNFYTRSAFLSAVNAYPG
FAHGGTEVEGKREIAAFFAHVTHETGHFCYISEINKSNAYCDASNRQWPCAAGQKYYGRG
PLQISWNYNYGPAGRDIGFNGLADPNRVAQDAVIAFKTALWFWMNNVHRVMPQGFGATIR
AINGALECNGNNPAQMNARVGYYKQYCQQLRVDPGPNLTC

>GRMZM2G117971
MAGMRVGKLALAAVLLCAAAAMATAQQASGVRATYNFYNPQQNNWDLNAVSAYCATWDAS
KPLSWRMKYGWTAFCGPAGPTGQAACGQCLLVTNTATGASITVRIVDQCSNGGLDLDYDT
AFKPIDTNGQGFQAGHLTVNYQFVNCGDN

>GRMZM2G010868P1
MARTQSAVAVAVVAAVLLLAAAATTSEAAITCGQVSSAIAPCLSYARGTGSGPSASCCSG
VRNLKSAASTAADRRAACNCLKNAARGVSGLNAGNAASIPSKCGVSIPYTISTSTDCSRVN

>GRMZM2G010868P2

MARTQSAVAVAVVAAVLLLAAAATTSEAAITCGQVSSAIAPCLSYARGTGSGPSASCCSG

VRNLKSAASTAADRRAACNCLKNAARGVSGLNAGNAASIPSKCGVSIPYTISTSTDCSR

>GRMZM2G101958

MARMQKLAVATAAVVALVLLAAAATSEAAISCGQVASAIAPCISYARGQGSGPSAGCCSG

VKSLNNAARTTADRRAACNCLKNAAAGVSGLNAGNAASIPSKCGVSIPYTISTSTDCSRVN

>GRMZM5G898755P1

MAARSSSSQPQLVAAAAVLAAALLLLAAGAGTASAAVSCGEVTSSVAPCLGYAMGSAASP

SAACCSGVRSLNSRASSTADRQATCNCLKSMTGRLGGGVSMANAANIPSKCGVSVGVPIS

PTVDCTKIN

>GRMZM5G898755P2

MAARSSSSQPQLVAAAAVLAAALLLLAAGAGTASAAVSCGEVTSSVAPCLGYAMGSAASP

SAACCSGVRSLNSRASSTADRQATCNCLKSMTGRLGGGVSMANAANIPSKCGVSVGVPIS

PTVDCTKYVGPAVPAPCMSS

>GRMZM2G107839P1

MAAVLNSRKTPQAVVAVLVAAALLASSASAAITCGQVGSSLAPCIPYATGRASALPASCC

SGVKSLNSAARTSADRQAACRCLKSLANSVKSVNMGTVATIPGKCGVSVGFPISMSTDCN

KYVIQLACLFAVIIHAYAYASPSFIFSILLGHACRIS

>GRMZM2G107839P2

MAAVLNSRKTPQAVVAVLVAAALLASSASAAITCGQVGSSLAPCIPYATGRASALPASCC

SGVKSLNSAARTSADRQAACRCLKSLANSVKSVNMGTVATIPGKCGVSVGFPISMSTDCNKIS

>GRMZM2G105364

MVTKVICFLVLASVLLAVAFPVSALRQQVKKGGGGEGGGGGSVSGSGGGNLNPWECSPKC

GSRCSKTQYRKACLTLCNKCCAKCLCVPPGFYGNKGACPCYNNWKTKEGGPKCP

>GRMZM2G068202

MAAASGRAPSACALLLLFLLLVVGAAAAAVIVVDANRGEQEQDWDWEQLSAASPSPWSPA

PAPAPSPVSFIDCGSACGARCALSSRWNLCRRACGSCCARCNCVPPGTAGNHDVCPCYAA

ITTRGGRPKCP

>GRMZM2G117940

MAQASSSFSIVLLFLALVLVVEVSAGTANEELYRPAGAEGSVPIEQCPEKCDYRCSATSY

KKPCLFFCNYCCNKCLCVPSGTYGNKEECPCYDNMKTKQGGPKCP

>GRMZM2G172596

MASRNKAAALLLCFLFLAAVAASAAEMIAGSGIGDGEGEELDKGGGGGGGHHKHEGYKNK

DGKGNLKPSQCGGECRRRCSKTHHKKPCLFFCNKCCAKCLCVPPGTYGNKETCPCYNNWK

TKKGGPKCP

>GRMZM2G062527P1

MESKSPWSLRLLICCAAMVAIALLPQQGGQAACFVPTPGPAPAPPGSSATNTNASSAAPR

PAKPSAFPPPMVSEYYR

>GRMZM2G062527P2

MESKSPWSLRLLICCAAMVAIALLPQQGGQAACFVPTPGPAPAPPGSSATNTNASSAAPR

PAKPSAFPPPMYGGVTPGTGSLQPHECGGRCAERCSATAYQKPCLFFCRKCCAACLCVPP

GTYGNKNTCPCYNNWKTKRGGPKCP

>GRMZM2G062527P3

MESKSPWSLRLLICCAAMVAIALLPQQGGQAACFVPTPGPAPAPPGSSATNTNASSAAPR

PAKPSAFPPPMYGGVTPGTGSLQPHGPYRTRL

>GRMZM2G062527P4

MYGGVTPGTGSLQPHGPYRTRL

>GRMZM2G062527P5

MYGGVTPGTGSLQPHECGGRCAERCSATAYQKPCLFFCRKCCAACLCVPPGTYGNKNTCP

CYNNWKTKRGGPKCP

>GRMZM2G077034

MKPAATARVAGLLFFLVLLLALPSLRVSMAGSGFCDGKCAVRCSKASRHDDCLKYCGICC

ATCNCVPSGTAGNKDECPCYRDMTTGHGNRTRPKCP

>GRMZM2G164090

MAKASSRLLFSLSLVVLLLLVETTTSPHGQADAIDCGASCSYRCSKSGRPKMCLRACGTC

CQRCGCVPPGTSGNEDVCPCYANMKTHDGQHKCP

>GRMZM2G107003

MAPSKLAVVVALVASLLLLTTSNTKLGLFVLGQAAPGAYPPRAPPPHQIVDLAKDCGGAC

DVRCGAHSRKNICTRACLKCCGVCRCVPAGTAGNQQTCGKCYTDWTTHGNKTKCP

>GRMZM2G032198

MESGSKKVAAGVLVLLLLQLMVAPTTATARLLQADTSPVFGLDFIAREFGHPDGAISCGE

SCVIIPCVSTLLGCRCENKLCVK

>GRMZM2G374405

MESGSKKVAACVLALLLLQLMVAPTTATARLLQQADTSPVFGLDIIAREFGHPDGAVPCF

ESCVFVPCISSVVGCRCENNVCVK

>GRMZM2G450866

MAARSPRGAVPDGSLATTPKVTMLSATLCYTGETCKYITCLTPACSLHPRTIPLLLLRRL

LGLPDLDRSVKIPPAVKACILDDAVARDIGVGQPLSNGRSSLLRSRVGGERFLWACCSGS

SSTTAGTSGIILTWHIATCILEVRHPYQCGGGGCQEQLNRHKIAATHLSRYCAYLMAWYP

DLLPDHEEWSTALYETVKDDARRALAGCSAGCAAVLGPEAEYVSRPVIQSLSVYSRHEGL

RKGVVLGKQLVELMEGEETAWMVLAEFWSEMILYVAASDIALHGVVS

>GRMZM2G430500

MAGRKCRAIPTAIATAVVVVAVLMAVAVAGVAAKDASGAPSSLTTWSAAGCSGDTSIVGS

CGCTDLAFYAGQEFYYRGETATLYTGTGCTGTPYQVFEDTQACGDFGWRSINIDC
