# Supplementary figures and images for: Investigation of Antimicrobial Peptide Genes Associated with Fungus and Insect Resistance in Maize
Source: Int J Mol Sci. 2017 Sep 15;18(9):1938. doi: 10.3390/ijms18091938 (PMC5618587; doi:10.3390/ijms18091938)

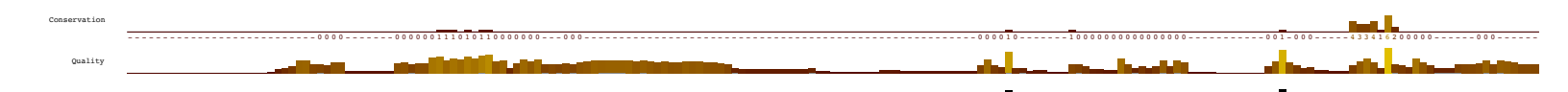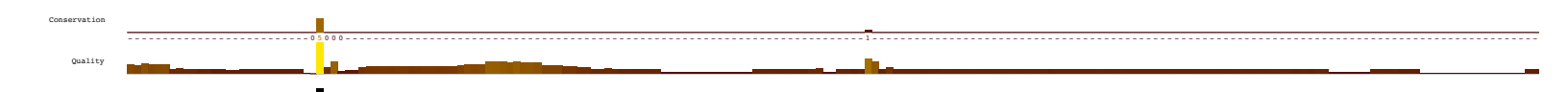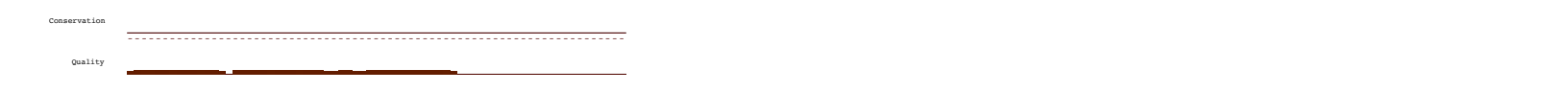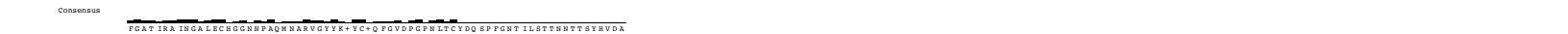

Supplement: Supplementary file 1 [file ijms-18-01938-s001.zip › Antimicrobial Peptide Genes in Maize_Supplemental Figure1.pdf]
